# Targeted Deletion of Sox10 by Wnt1-cre Defects Neuronal Migration and Projection in the Mouse Inner Ear

**DOI:** 10.1371/journal.pone.0094580

**Published:** 2014-04-09

**Authors:** YanYan Mao, Simone Reiprich, Michael Wegner, Bernd Fritzsch

**Affiliations:** 1 Department of Biology, University of Iowa, Iowa City, Iowa, United States of America; 2 Otolaryngology–Head and Neck Surgery, Provincial Hospital affiliated to Shandong University, Jinan, China; 3 Institute for Biochemistry, University of Erlangen, Erlangen, Germany; Universitat Pompeu Fabra, Spain

## Abstract

Sensory nerves of the brainstem are mostly composed of placode-derived neurons, neural crest-derived neurons and neural crest-derived Schwann cells. This mixed origin of cells has made it difficult to dissect interdependence for fiber guidance. Inner ear-derived neurons are known to connect to the brain after delayed loss of Schwann cells in ErbB2 mutants. However, the ErbB2 mutant related alterations in the ear and the brain compound interpretation of the data. We present here a new model to evaluate exclusively the effect of Schwann cell loss on inner ear innervation. Conditional deletion of the neural crest specific transcription factor, Sox10, using the rhombic lip/neural crest specific Wnt1-cre driver spares Sox10 expression in the ear. We confirm that neural crest-derived cells provide a stop signal for migrating spiral ganglion neurons. In the absence of Schwann cells, spiral ganglion neurons migrate into the center of the cochlea and even out of the ear toward the brain. Spiral ganglion neuron afferent processes reach the organ of Corti, but many afferent fibers bypass the organ of Corti to enter the lateral wall of the cochlea. In contrast to this peripheral disorganization, the central projection to cochlear nuclei is normal. Compared to ErbB2 mutants, conditional Sox10 mutants have limited cell death in spiral ganglion neurons, indicating that the absence of Schwann cells alone contributes little to the embryonic survival of neurons. These data suggest that neural crest-derived cells are dispensable for all central and some peripheral targeting of inner ear neurons. However, Schwann cells provide a stop signal for migratory spiral ganglion neurons and facilitate proper targeting of the organ of Corti by spiral ganglion afferents.

## Introduction

Whereas spinal sensory neurons are purely neural crest-derived, most cranial nerves of vertebrates are composed of a mixture of neural crest-derived sensory neurons, neural crest-derived Schwann cells and a variable contribution of placode-derived sensory neurons as well as branchial motor axons, a mix that arose early in chordate evolution [1]–[3]. Multiple attempts have been made to sort out the relative importance of neural crest-derived cells for the guidance of central and peripheral projections of placode-derived neurons and for branchial motoneuron axons extending along them. Extirpation of neural crest suggested that proximal projections of sensory neurons require neural-crest-derived cells [4]. Consistent with this suggestion is that removal of placodes only mildly disrupts delaminating neural crest cell migration, whereas altered trajectories of neural crest cell migration redirects projections of placode-derived neurons [5]. In agreement with these experimental data in chicken, lack of Schwann cell differentiation in ErbB2 mutants leads to peripheral nerve mistargeting in mice [6], including disoriented and reduced projection to the cochlea and cochlear nucleus [7].

In contrast to these experimental data in chicken and some mammals that strongly suggest a role of neural-crest-derived cells for placode-derived neuronal fiber guidance, other data in mammals suggest limited effects of neural crest-derived cells on placode-derived neuronal projections. For example, a Wnt1-cre mediated diphtheria toxin ablation of neural crest cells in mice showed normal peripheral epibranchial nerve fiber projections despite absence of Sox10 positive cells [8]. Consistent with this work on epibranchial placode- derived neurons, work on Sox10 mutant mice suggested that the equally placode-derived inner ear neurons develop completely normal in the absence of Sox10 dependent neural crest cells [9], [10]. The latter papers are difficult to reconcile with the fact that Sox10 is not only expressed in neural crest but also in the ear [11]. Much like ErbB2, Sox10 could possibly affect the development of inner ear connections both through Schwann cells and through direct effects in the ear. Because of these dual actions in Schwann cells and the ear, it remains unclear how much neural crest-derived Schwann cells alone contribute to pathfinding in the ear [7], [10].

Sox10 is a member of the SoxE family of transcription factors (Sox8-10) that are characterized by the conserved SRY-like HMG box [12], [13]. Sox10 is expressed in all neural crest cells and is especially crucial for specification, survival, and differentiation of Schwann cells derived from neural crest stem cells [14], [15] through activation of major genes needed for Schwann cell development such as ErbBs and Krox20 [16]. After delaminating from the dorsal neural tube, neural crest cells migrate to various organs and differentiate into pigment cells, Schwann cells [17], [18] and most neurons of the PNS, except for placode-derived neurons [2], [3], [5], . In the mammalian inner ear, the intermediate pigment cells of the stria vascularis and Schwann cells derive from neural crest [10]. Absence of these pigment cells leads to Waardenburg syndrome (WS) and Sox10 mutations are associated with both WSII and WSIV [21]–[23]. In addition to neural crest, Sox10 (and also Sox9) are expressed early and widespread in the ear [11], [24]. Overexpression of either Sox9 or Sox10 in *Xenopus* results in ectopic otocysts [25] indicating an important function of Sox10 in ear development in addition to the essential role in neural crest development.

Given the known and potential function of Sox10 in both neural crest and ear development [7], [14], [25], we used Wnt1-cre to conditionally delete Sox10 in the neural crest alone, sparing the expression of Sox10 in the ear. We achieved this by crossing a floxed Sox10 line [12] with a Tg(Wnt1-cre) line [26]. We chose this approach over the Wnt1-cre induced diphtheria toxin to sidestep the previously reported potential late replenishment of neural crest [8]. We show that spiral ganglion neurons (SGN) migrate beyond their normal position toward the brain, comparable to vestibular ganglion neurons. Fibers in the ear reach the organ of Corti but also project to the lateral wall with no obvious correction over time.

## Materials and Methods

### Mice and Genotyping

Sox10 mutant mice suffer from multiple organ syndrome and die around E14 [14]. In order to avoid this problem and effects of Sox10 in the ear, we used *Sox10^f/f^*, Tg(Wnt1-cre) mice. The Wnt1 gene is expressed specifically in the neural plate, in the dorsal neural tube and in the early migratory neural crest population at all axial levels excluding the forebrain [26], [27]. Expression of Wnt1 is extinguished as the crest cell lineage migrates away from the neural tube, and is not expressed at any other time or in any other place during development or in postnatal life [28]. Wnt1-cre has been previously used to eliminate neural crest at least transiently by expressing diphtheria toxins [8]. We used this Cre line to recombine the neural crest-expressed floxed Sox10 [12].

Pregnant females were sacrificed under isoflurane anesthesia and embryos dissected in ice-cold PBS followed by immediate decapitation. Embryos were dissected from anesthetized pregnant females at embryonic days 12–16, counting noon of the day of the vaginal plug was found as E0.5. Embryos were fixed in 4% paraformaldehyde (PFA) in 0.1 M phosphate buffer (pH 7.4). Some older embryos were fixed with 4% PFA and 1% glutaraldehyde in 0.1 M phosphate buffer for electron microscopy analysis. All of the embryos were genotyped by polymerase chain reaction analysis of tail DNA by using cre-specific primers and Sox10- specific primers. We used a total of 26 conditional Sox10 mutant embryos (Sox10 CKO; E12.5–18.5) and an equal number of littermate controls. We define as littermate control mice that either have no Wnt1-cre or no floxed Sox10 gene. Heads fixed in 4% paraformaldehyde (PFA in 0.1 M phosphate buffer (pH 7.4) were shipped to the University of Iowa for analysis of ear defects. Animals were originally coded for double blind assessment of the phenotype. Given the clear phenotype, genotypes were disclosed with later shipments. Only aldehyde fixed tissue was processed at the University of Iowa. Processing of preserved tissue does not require IACUC approval.

For direct comparisons of the effects of Schwann cell removal on the ear innervation we added limited previously unpublished data from work done on ErbB2 mutants [7].

### Ethics Statement

All animal procedures were handled at the University of Erlangen in accord with German Animal Care laws avoiding any undue suffering of animals. Animals were housed in approved cages with regular access to food and water and appropriate day/night cycle. All animal procedures were registered, and approved by the responsible governmental agency of Mittelfranken.

### In situ Hybridization

Whole mount *in situ* hybridization was conducted using digoxigenin-labeled RNA probes. Antisense RNA probes were generated from plasmids containing the cDNAs and in vitro transcription was carried out to label the probe with digoxigenin by DIG RNA labeling kit (Roche Applied Science, Cat.11175025910). The ears were dissected in 0.4% PFA under RNase-free conditions, dehydrated and rehydrated in a graded methanol series, and digested briefly with 20 mg/ml of Proteinase K (Ambion, Austin, TX, USA) for 15–20 min. The samples were then hybridized overnight at 60°C to the riboprobe in hybridization solution containing 50% (v/v) formamide, 50% (v/v) 2× saline sodium citrate (Roche), and 6% (w/v) dextran sulfate. After washing off the unbound probe, the samples were incubated overnight with an anti-digoxigenin antibody (Roche Diagnostics GmbH, Mannheim, Germany). After several washes, the samples were incubated with nitroblue phosphate/5-bromo, 4-chloro, 3-indolil phosphate (BM purple substrate, Roche Diagnostics GmbH, Mannheim, Germany), which is enzymatically converted to a purple colored product. The ears were flat-mounted in glycerol and imaged in a Nikon Eclipse 800 microscope using differential interference contrast microscopy and Metamorph software. For each *in situ* hybridization experiment at least two ears were analyzed at a given stage. However, a given ear was used for multiple analysis such as tract tracing, in situ hybridization, immunocytochemistry and histology following a published protocol [29]. We analyzed the following gene expression: Sox10, Mmp-24 (previously MT5-MMP) and Ntf3 (previously NT-3).

### Plastic Embedding and Stevenel’s Blue Staining

For sections, ears were dissected, washed in 0.1 M phosphate buffer, dehydrated in graded ethanol, immersed in propylene oxide, embedded in Epon 812, and incubated at 60°C oven for at least 24 hours. 0.5 to 2 μm thick sections were cut using a Leica RM2265 Microtome with diamond knife and sections were mounted on slides. Some sections were stained with Stevenel’s blue and imaged by a Nikon Eclipse 800 microscope and Leica TCS SP5 confocal microscope. Comparable sections were available from a previous study on ErbB2 null mutants [7].

### Immunohistochemistry and Apoptotic Cell Labeling

For immunohistochemistry staining, the ears were dissected, dehydrated in 100% ethanol and then rehydrated in graded ethanol series. After washing in phosphate-buffered saline (PBS), they were blocked with 2.5% normal goat serum in PBS containing 0.5% Triton-X-100 for 1 hour. The primary antibodies for Myo7a (Myosin 7a, Proteus Biosciences), Tubulin (Sigma), Neurofilament (Millipore), activated Caspase3 (Cell Signaling Technology), were diluted in block buffer for 1∶100, 1∶800, 1∶1000, 1∶100 respectively. The samples were washed in PBS for 1 hour and incubated in one or a combination of diluted primary antibodies for 48–72 hours on a shaker at 4°C. Corresponding secondary antibodies diluted in block buffer (1∶500) (Alexa fluor molecular probe 647 or 532 or 488; Invitrogen) were added and incubated overnight at 4°C. The PSVue480 (10 mM; Molecular Targeting Technologies, Inc., P-1003) and Hoechst (10 mg/mL; Polysciences, Inc., 09460) were incubated for 1 hour at room temperature. After repeated PBS washes, the ears were mounted in glycerol and imaged by a Leica TCS SP5 confocal microscope.

### Lipophilic Dye Tracing

The fixed heads were hemisected along the midline. Two differently colored lipophilic dye-soaked filter strips [30] were inserted into the alar plate of rhombomere 6 (NeuroVue red) and basal plate of rhombomere 4 (NeuroVue maroon) in order to label the afferent and efferent fibers of the ear, respectively, as previously described [7]. For central dye tracing from the ear to the brain, NeuroVue red dye and NeuroVue maroon were inserted into base and apex of the cochlea, respectively. After the injection, the half heads were put in the oven at 60°C in 4% PFA for 24–72 h depending on the stages of mice for proper diffusion. The inner ears and brains were then dissected out, mounted on glass slides in glycerol, and viewed immediately by a Leica TCS SP5 confocal microscope. Confocal stacks were collapsed along the Z-axis to generate single focus images.

### Measurements of Distance of Spiral Ganglion Neurons from the Cochlear Duct

As in a previous study of mutant mice without Schwann cells [7], our data showed unusual positions of spiral ganglion neurons. We quantified this phenomenon by measuring the distance of spiral ganglion neurons to the medial wall of the cochlear duct in the middle turn of five whole mounted base-to-middle turn cochleae stained for spiral ganglion neurons using either p75^NTR^ or Mmp-24 *in situ*. To avoid distortions in the mounting procedure, we always co-mounted Sox10 mutant and control littermates and measured at the same microscope magnification the distance.

### Quantification of Apoptotic Profiles

Previous work on mice without Schwann cells [7] suggested profound loss of neurons via apoptosis in the absence of Schwann cells and altered gene expression in the ear. Our data using apoptotic markers identified few apoptotic cells consistent with previous reports on the developing normal mouse ear [31], [32]. The apparent mixing of spiral and vestibular neurons outside the ear blocked us from counting the total of remaining spiral ganglion neurons. Instead, we determined the ratio of apoptotic to normal neurons, counting only neurons inside the ear as we had no evidence of mixing of vestibular and spiral ganglion neurons inside the ear. Some techniques to image apoptotic cells appear to be unreliable in the ear [33] but we could confirm some overlap of apoptotic profiles with more recently developed techniques to label apoptotic cells [34]. Apoptosis can be easily visualized using plastic embedding which also avoids false negative results due to lack of experimental labeling of apoptotic profiles. We counted numbers of apoptotic profiles in two non-consecutive 2 μm thick epoxy sections about 25 μm apart of ears taken from two Sox10 conditional deletion mice, two Sox10 control littermates and two ErbB2 mutants from previous work [7] at two stages (E16.5; E18.5). Means were computed and a ratio of apoptotic profiles to normal spiral ganglion neurons was calculated to establish the relative frequency of apoptotic profiles (Table 1). Data from the two stages were combined and differences in the frequency of apoptotic profiles were tested in Sox10 conditional mutant and ErbB2 mutants for significance using the Mann-Whitney U-test [35] and accepted at the p = 0.05 level. Pooled data were also present as a simple diagram to indicate the overall more profound apoptosis in ErbB2 mutants compared to Sox10 conditional mutants and Sox10 control littermates.

**Table 1:** Apoptotic profiles in control, Sox10 and ErbB2 mutants.

	ErbB2^−/−^ E16.5 (N = 2),18.5 (N = 2)	Wnt1-cre; Sox10^f/f^ E16.5 (N = 2),18.5 (N = 2)	Control E16.5 (N = 2), 18.5 (N = 2)
#normal SGNs per area	E16.5∶470, 358E18.5∶205,353	E16.5∶396, 380E18.5∶389, 365	E16.5∶444, 405E18.5∶415, 390
SGN average per area	347	383	414
Standard deviation	77	17	23
# of apoptotic profiles per area	E16.5∶58, 85E18.5∶67, 87	E16.5∶11,12 E18.5∶12, 14	E16.5∶6, 5E18.5∶4, 2
apoptotic average	74	12	4
Standard deviation	22	3	1
ratio of apoptotic profiles per area	E16.5∶12,24E18.5∶18, 25	E16.5∶3, 3E18.5∶3, 4	E16.5∶1,2E18.5∶1,1
ratio average	20*	3*	1
Standard deviation	8	1	0.5

*Indicates significant difference between the ratio of apoptotic profiles in Sox10 mutant and ErbB2 mutants (p = 0.05), pooling all samples across two stages. Mann-Whitney U-test [35].

# mean of two sections per each of two distinct samples.

## Results

### Verification of Complete Recombination of Sox10

**Figure 1:**
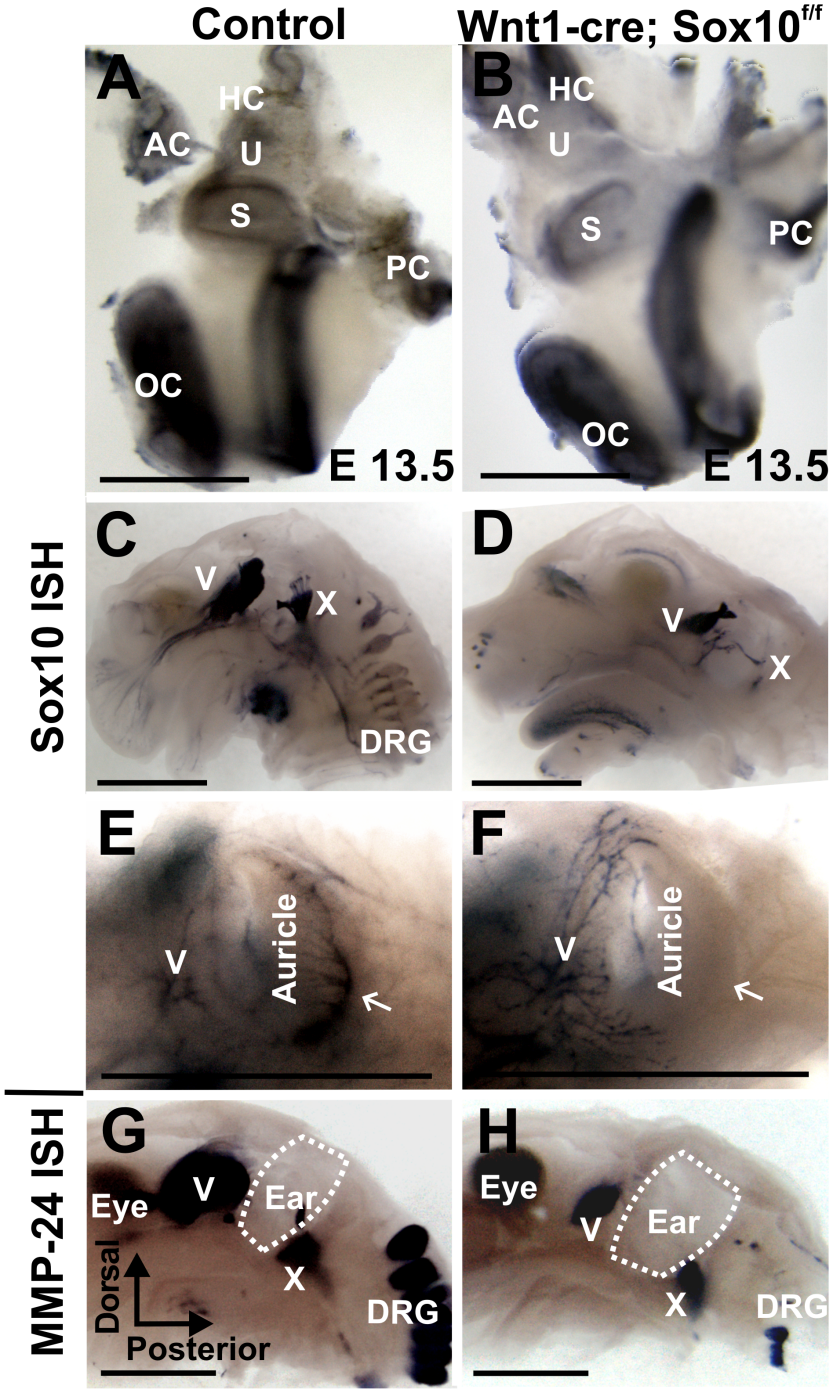
Conditional deletion of Sox10 with Wnt1-cre eliminates Sox10 expression in neural crest but not placode-derived cells. These images of E13.5 mice show that Sox10 expression is retained in the ear (A, B) and some other cranial placode-derived ganglia (C, D). Sox10 is in the Schwann cells of spinal dorsal root ganglia (DRG) in the control (C) but no cervical DRGs are Sox10 positive in the Wnt1-cre; Sox10 conditional mutant mice (D). Trigeminal ganglion neurons (V) innervate the anterior part of the ear lobes (E, F) whereas the posterior aspect of the ear lobes is innervated by the greater auricular nerve of the DRGs in control animals (arrow in E), but not in the mutants (arrow in F). Mmp-24 is an early neuronal marker and *in situ* hybridization (G, H) shows reduction of the remaining ganglia (G, H). Some Mmp-24 positive DRG neurons but no autonomic ganglia remain in Sox10 mutants (H). Abbreviations: OC, organ of Corti; PC, posterior crista; AC, anterior crista; HC, horizontal crista; U, utricle; S, saccule; DRG, dorsal root ganglia. Dotted line indicates where the ear was removed in G, H; bar indicates 0.5 mm in A, B, and 1 mm in C–H.

While previous work has demonstrated that Wnt1-cre can effectively label and, using diphtheria toxin, eliminate all neural crest cells [8] there are discrepancies in the literature about viability of sensory neurons (possibly dying directly or as a consequence of absence of Schwann cells) and the rostro-caudal efficacy of Sox10 elimination [22]. Previous work has suggested that many neural crest-derived cells labeled with Wnt1-cre become incorporated into the ear including the sensory epithelia [36], but more recent work failed to confirm this claim [20] or questioned it [3]. Given these discrepancies, we wanted to verify that Wnt1-cre does not disrupt the Sox10 expression in the ear due to incorporation of Sox10 negative cells from the neural crest. Using Sox10 *in situ* hybridization we verified that at E13.5 the Sox10 expression was similar in the mutant ear relative to control (Fig. 1A, B) with no obvious gaps in sensory epithelia formation. At the level of the whole mount our data showed no Wnt1-cre mediated depletion of Sox10 in the ear as implied in one previous paper [36] but questioned in a more recent publication [20]. Further below we will provide more detailed histology of normal sensory epithelia development consistent with our interpretation of the *in situ* hybridization data.

We next investigated the development of other cranial and cervical spinal nerves in this Sox10 conditional mutant line to identify if there is complete loss of Sox10 expression, except for placode-derived ganglia [2]. In control animals, Sox10 expression was particularly profound throughout the trigeminal (V) and glossopharyngeal/vagal ganglion (IX/X) complex (Fig. 1 C). However, cervical spinal ganglia showed only the characteristic spotted expression distribution in the nerves and ganglion consistent with Sox10 expression in Schwann cell precursors. In the Sox10 conditional mutant we found uniform Sox10 labeling in the smaller trigeminal and glossopharyngeal/vagal ganglion (Fig. 1D). We could not identify any Sox10 expressing cervical dorsal root ganglia (Fig. 1D). In contrast to control animals (Fig. 1E), Schwann cells were absent in the cervical spinal nerves in Sox10 conditional mutants (Fig. 1D) including the greater auricular nerve (Fig. 1F) innervating the posterior aspect of the developing external ear. The anterior part of the ear, innervated by the trigeminal auriculo-temporal nerve, showed no discernible differences in our conditional mutants compared to control littermates, indicating near normal presence of Schwann cells (Fig. 1E, F).

These data suggest that Wnt1-cre mediated Sox10 deletion eliminates spinal but incompletely eliminates cranial neural crest cells and spares placode-derived neurons associated with the trigeminal and glossopharyngeal/vagal ganglia (Fig. 1C, D). Therefore, at least some cranial and spinal ganglia remain in the conditional deletion model. We next investigated those neurons using a marker for early delaminating neural crest neurons [37], the metalloproteinase Mmp-24 [previously MT5-MMP [38]]. Our data revealed smaller neuronal populations in most cranial and spinal sensory ganglia (Fig. 1G, H), indicating the expected sparing not only of placode-derived neurons but also of some cervical dorsal root ganglia, a neural crest-derived neuronal population.

**Figure 2:**
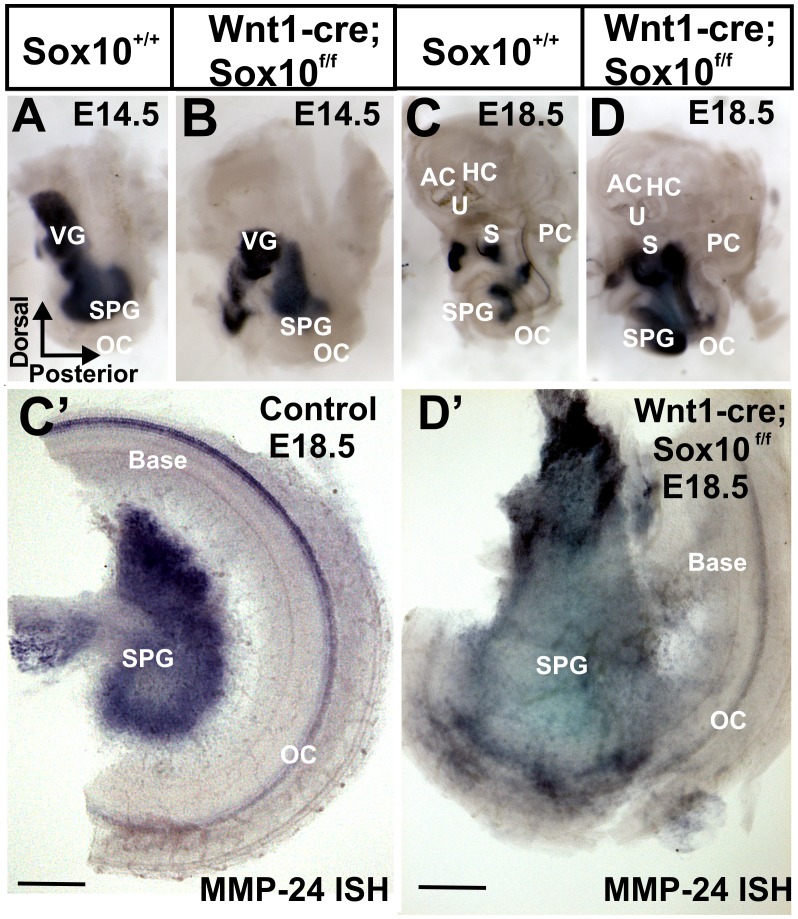
Development of spiral ganglion neurons as revealed with Mmp-24 *in situ* hybridization. The metalloproteinase Mmp-24 is an early marker for sensory neurons of the ear (A–D) and also labels the organ of Corti at later stages (C, D). This marker reveals that developing spiral ganglion neurons are differently distributed inside the developing cochlea from E14.5 (A, B) to E18.5 (C, D): Instead of dispersing along the cochlea (A, C) neurons lump together in the center of the cochlea (B, D). Whereas spiral ganglion neurons are in control mice at a nearly constant distance from the cochlea (C’) all spiral ganglion neurons aggregate in the center of the basal turn in the Wnt1-cre;Sox10^f/f^ littermates (D’). Abbreviations: OC, organ of Corti; PC, posterior crista; AC, anterior crista; HC, horizontal crista; U, utricle; S, saccule; SPG, spiral ganglion; VG, vestibular ganglion. Bar indicates 100 μm.

We next investigated the development of inner ear neurons using Mmp-24 in situ hybridization, apparently a reliable marker for all early delaminating neurons (Fig. 2). We could not detect any difference at E12.5 but found a very different distribution of neurons in the ear in E14.5 and E18.5 Sox10 mutants. Whereas spiral ganglion neurons formed a spiral in parallel to the cochlear duct (Fig. 2A, C), in Sox10 mutants these neurons tended to be grouped together in the center of the basal turn (Fig. 2B, D). We next split the cochlea into a basal and apical half to provide a clearer image of the unusual distribution of spiral ganglion neurons. In control animals, spiral ganglion cells were indeed parallel to the Mmp-24 positive cochlear duct (Fig. 2C’). In contrast, nearly all spiral ganglion neurons accumulated in the center of the basal turn in Sox10 conditional mutants (Fig. 2D’).

**Figure 3:**
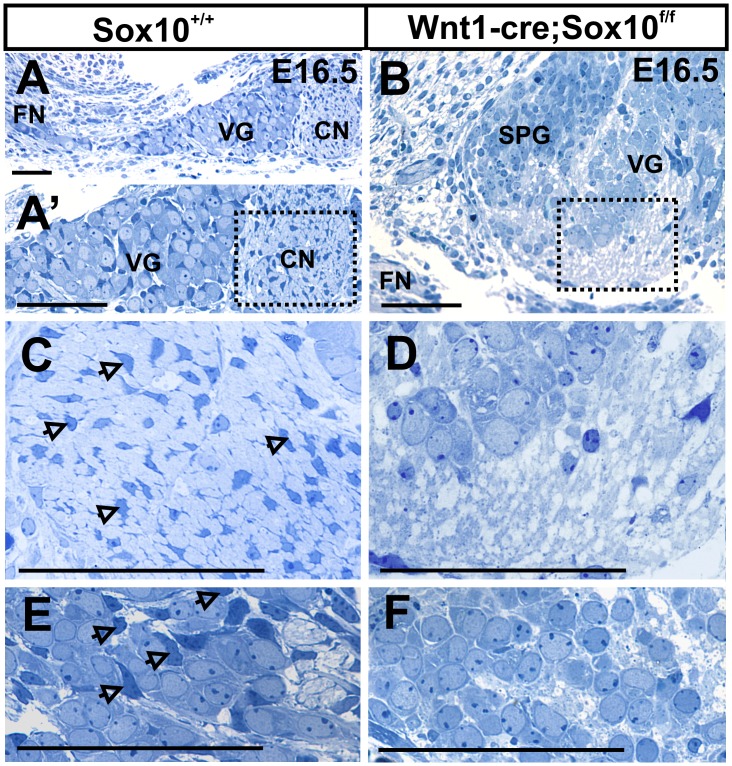
Sox10 conditional mutants have no Schwann cells and show different distribution of neurons. These sections are cut parallel to the facial nerve (FN) to obtain cross sections through the vestibular (Scarpa’s) ganglion and cochlear nerve between the ear and the brain. Vestibular ganglion (VG) neurons are anterior and adjacent to the cochlear nerve (CN) in control mice (A, A’). In Sox10 mutants, vestibular and spiral ganglion neurons co-mingle next to the facial nerve (B). Schwann cells with small, elongated and heterochromatin rich nuclei are readily visible between nerve fibers (arrows in C) and the developing spiral ganglion (E) in control mice. No such cells exist in the Sox10 mutants (D, F). Abbreviations: CN, cochlear nerve; FN, facial nerve; SPG; spiral ganglion; VG, vestibular ganglion. Bar indicates 100 um in A–F.

Sections through the control vestibular ganglion, located between the ear and the brain, showed the well-known organization of a cochlear nerve immediately adjacent and caudal to the vestibular (Scarpa’s) ganglion (Fig. 3A, A’). In contrast, in Sox10 mutants we found a mixture of what appeared to be spiral and vestibular ganglion neurons next to the facial nerve between the ear and the brain (Fig. 3B). These data suggest that spiral ganglion neurons are abnormally distributed in the Sox10 conditional mutants.

Since the changed distribution of spiral ganglion neurons was reminiscent of a previous report in which the absence of Schwann cells was directly visualized using a fluorescent reporter [7] we next wanted to verify the loss of Schwann cells in the developing spiral ganglion of Sox10 conditional deletion mice. While several markers suggested absence of Schwann cells (data not shown) we were concerned that Schwann cell markers may be unreliable due to the conditional Sox10 elimination, providing false negative to incompletely differentiated cells. To avoid such possible false negatives, we histologically verified the absence of Schwann cells at the light microscopic level. Semithick epoxy resin sections showed no cells bearing the cytological characteristics of Schwann cells in either the ganglia (Fig. 3E, F) or the nerve (Fig. 3C, D).

In summary, our data reveal no Schwann cells in the ganglia or the inner ear nerves in Sox10 conditional mutants but indicate an unusual and mixed distribution of spiral and vestibular neurons near the facial nerve (Fig. 3B, D, F). These data are consistent with previous suggestions of migratory defects in sensory neurons in the absence of ErbB2 mediated differentiation of Schwann cells [7], possibly related to a stop signal provided by Schwann cells [39].

### Absence of Schwann Cells Results in Additional Migration of Spiral Ganglia

**Figure 4:**
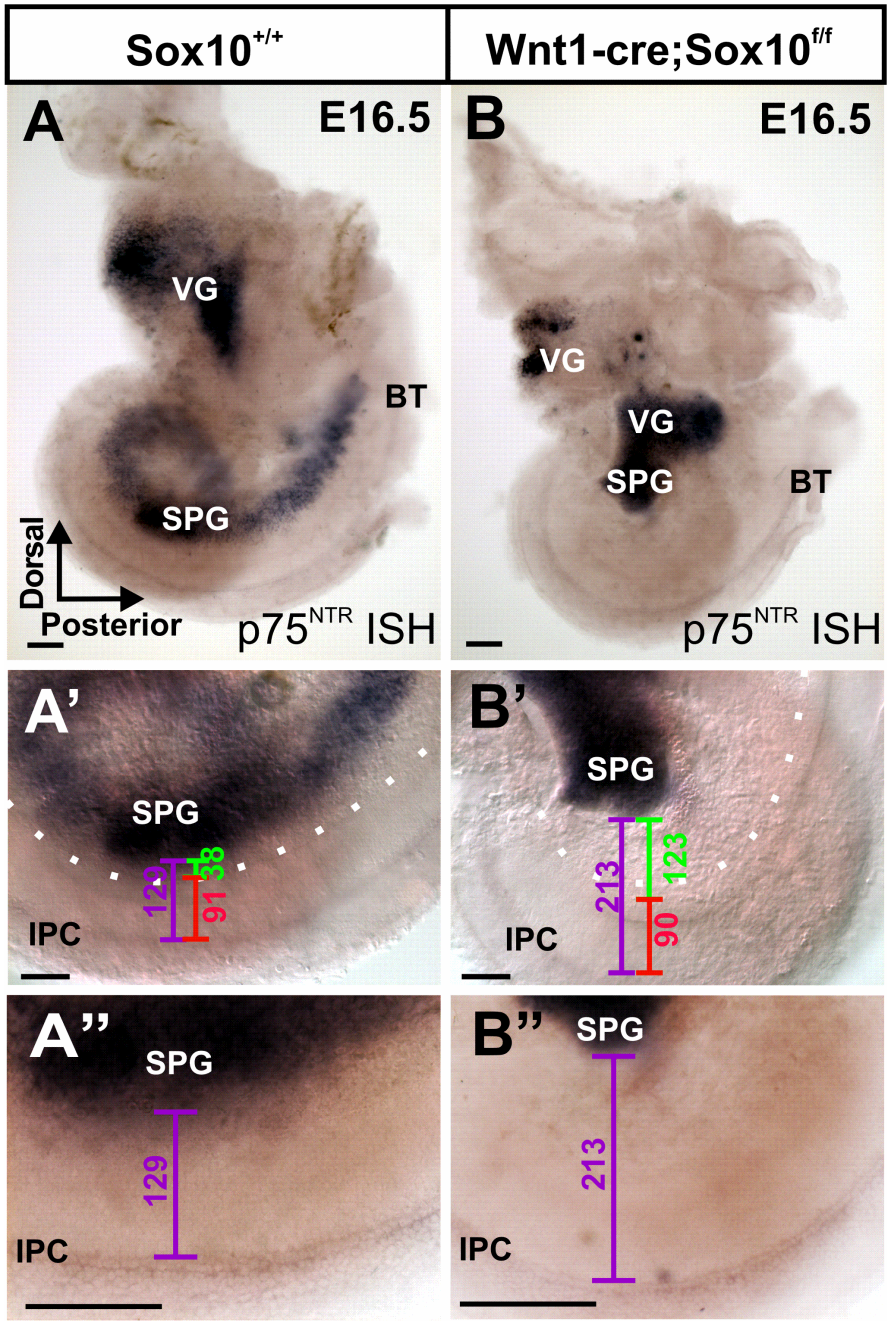
Conditional Sox10 mutants reveal differential migration of spiral ganglion neurons. p75^NTR^ *in situ* hybridization labels all sensory neurons of the ear and the inner pillar cells of the organ of Corti [40]. Labeling both cell types with a single marker allows for measuring the distance between the spiral ganglion neurons (SPG) and the inner pillar cells (IPC) (lilac bar in A’, B’, A”, B”). Note the altered ganglion cell distribution (A, B) and the 4 times increase in distance between the SPG and the cochlear duct (green bar), but constant distance to the inner pillar cells (red bar) in these littermates. Abbreviations: BT, basal tip; VG, vestibular ganglion; U, utricle; SPG, spiral ganglion neurons; IPC, inner pillar cells. Bar indicates 100 um.

We next aimed to quantify this differential migration effect using markers that co-label the sensory neurons and the organ of Corti throughout development. The neurotrophin receptor p75^NTR^ is expressed in delaminating sensory neurons and the single row of inner pillar cells extending along the organ of Corti [40]. This marker allows quantifying possible migratory effects at a cellular resolution level. Consistent with past reports on the distribution of spiral ganglion neurons in control animals, our data showed the arrangement of spiral ganglion neurons in parallel and at a nearly constant distance to the spiraling cochlear duct and inner pillar cells of the organ of Corti (Fig. 4A). In contrast, the Sox10 conditional deletion mice had all spiral ganglion neurons lumped in the center of the cochlear spiral and noticeably further away from the inner pillar cells of the organ of Corti (Fig. 4B). Most importantly, while the distance from the medial cochlear wall to the inner pillar cells was identical in control and mutant mice (red line in Figs. 4A’, B’), the distance between the spiral ganglion neurons and the cochlear medial wall was dramatically increased (green line in Fig. 4A’, A”, B’, B”) suggesting that the differences are not due to a mounting artifact leading to differential compression. As previously suggested in ErbB2 null mutants [7], differentiated Schwann cells may provide a molecularly unknown signal that stops migration of spiral ganglion neurons as they delaminate from the ear. In the complete absence of any Schwann cells (this study) or of differentiated Schwann cells [7] spiral ganglion neurons migrate into the center of the ear (Fig. 4B) and toward the brain, much like vestibular neurons (Fig. 3B).

We measured the distance between the spiral ganglion and the medial wall of the cochlear duct in 5 ears reacted with either Mmp-24 (Fig. 2) or p75^NTR^ (Fig. 4). In these five E18.5 day old ears, we found that spiral ganglia in the middle turn were about 4 times further away (green lines in Fig. 4A’, B’) from the medial wall of the cochlear duct in Sox10 mutants (Mean = 125 μm; St. Dev = 28; N = 5) compared to control littermates (Mean = 34 μm; St. Dev = 14; N = 5). This indicates that absence of Schwann cells causes an approximately 3–4 times additional migration of spiral ganglion neurons.

### Conditional Deletion of Sox10 Results in Aberrant Projections that Bypass the Organ of Corti

**Figure 5:**
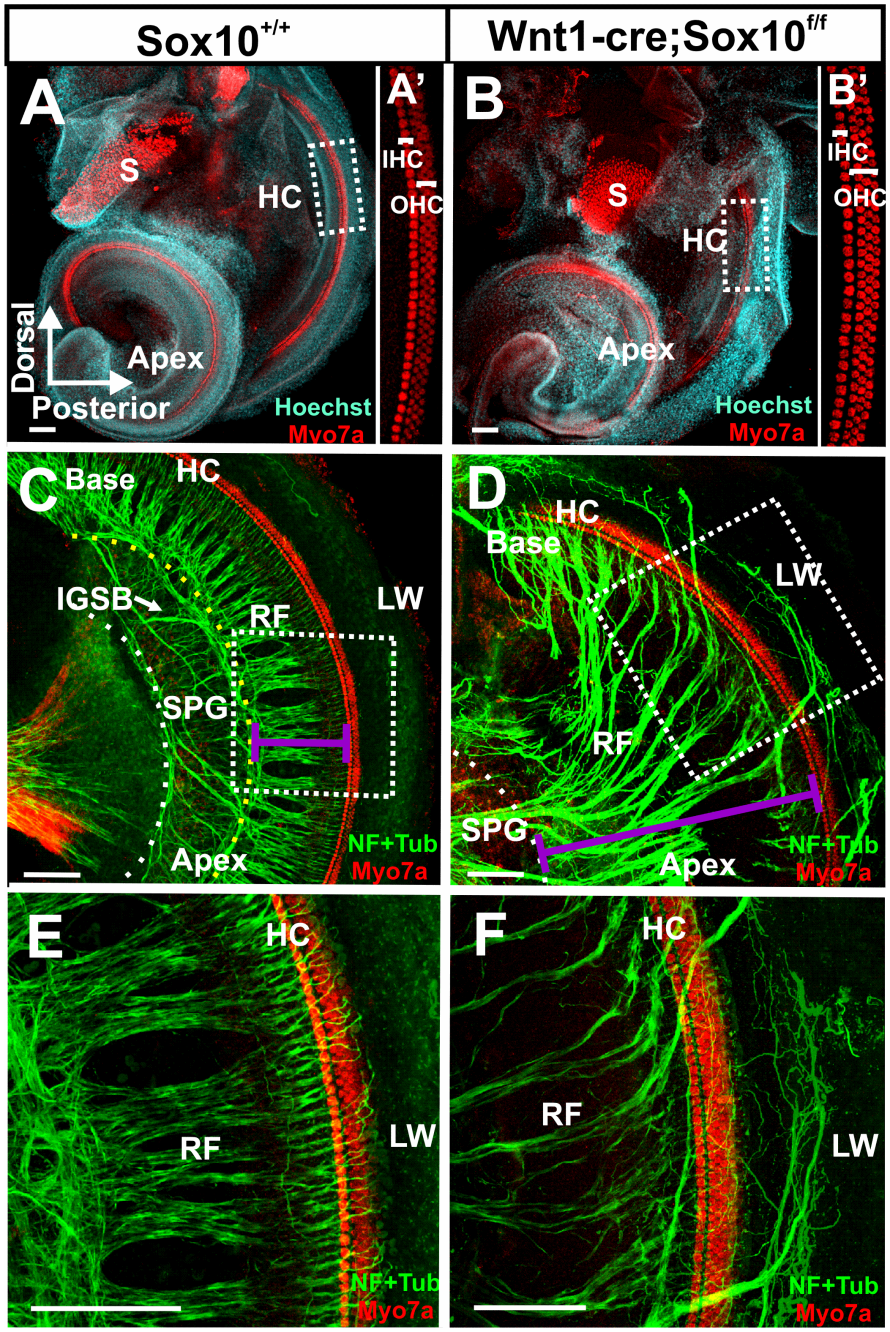
Sox10 mutants have unusual innervation patterns. Immunocytochemistry of Sox10 conditional knockout mice show normal organization of organ of Corti hair cells (A, A,’B, B’). Myo7a (red) labels one row of inner hair cells (IHC) and three rows of outer hair cells (OHC; A’, B’). Short radial fibers (RF) project from spiral ganglion neurons (SPG) to the organ of Corti (lilac line in C), indicated by the Myo7a labeled hair cells (HC in C, E). In contrast, Sox10 conditional mutants have elongated radial fibers (lilac line in D) and have no intraganglionic spiral bundle (IGSB) formed by efferent axons in the displaced spiral ganglion (C, D). The regular spacing of radial bundles is disrupted in the Sox10 mutant mice and fibers overshoot the organ of Corti hair cells (HC) to end in the lateral wall (LW in E, F). Abbreviations: HC, hair cells; RF, radial fibers; IGSB, intraganglionic spiral bundle; SPG, spiral ganglion; S, saccule; LW, lateral wall. Bar indicates100 μm.

Our data show that the absence of Sox10 positive neural crest-derived cells results in altered migration of spiral ganglion neurons comparable to ErbB2 mutant mice. We next investigated if the peripheral projection to the inner ear is as affected as in ErbB2 mutants [7]. We first verified that all hair cells were normally developed (Fig. 5A, B) as previous work had claimed that a variable number of hair cells is derived from neural crest [36] and could thus potentially be missing, defecting innervation as previously described [41]. Our data revealed no effect of Wnt1-cre mediated ablation of Sox10 on hair cells in the organ of Corti (Fig. 5A, B) or other sensory epithelia of the ear, consistent with recent experimental tracing show that neural crest provides only non-neurosensory cells for the ear [20]. Despite the unusual position of spiral ganglion neurons in the Sox10 conditional mutants (Fig. 4), the innervation of the cochlea was remarkably normal (Fig. 5C–F), with a few noticeable exceptions. In the more developed basal turn, bundles of fibers were found to bypass the organ of Corti to extent to the lateral wall in the mutants (Fig. 5D, F). The two following changes were most obvious: radial fibers did not have the regular spacing found in control animals and there were no intraganglionic spiral bundles (IGSB) of efferents (Fig. 5C, F). The phenotype conformed closely to the previous description in ErbB2 mutant mice [7], suggesting that the deviations from normal projections are largely related to aberrant pathfinding of nerve fibers and aberrant migration in the absence of Schwann cells.

Using neurofilament and tubulin immunocytochemistry we found that radial bundles are at E18.5 at least three times the length in the Sox10 conditional mutant (467 um +- 12; N = 3) of control littermates (157 um +- 8; N = 3; lilac lines in Fig. 5C, D). These are similar overall changes compared to whole mounts of *in situ* labeled neurons (Fig. 4A, B). Obviously, processes of spiral ganglion neurons have to cover a longer distance to reach from the displaced spiral ganglion neurons to organ of Corti, thus lengthening greatly the radial fibers.

**Figure 6:**
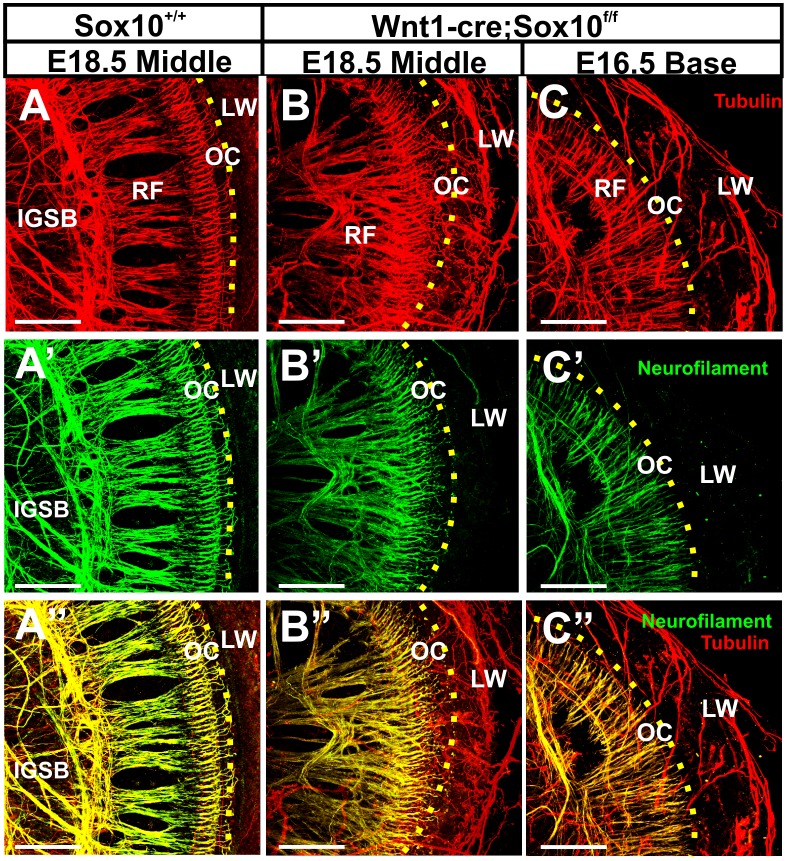
Sox10 CKO mice show unusual distribution of anti-tubulin labeled fibers. In the control mice, neurofilament and tubulin double labeled fibers (A,A’, A”) extend in radial fiber bundles to the hair cells of the organ of Corti (indicated by yellow dotted line). In contrast, in Sox10 conditional mutants radial fibers are disorganized as revealed by either anti-tubulin (B, C) or anti-neurofilament (B’, C’). Superimposing the double labeling shows nearly complete overlap of fiber staining with either antibody in control (A”) but segregation of many tubulin labeled fibers in Sox10 mutants, in particular those to the lateral wall (B”, C”). Abbreviations: IGSB, intraganglionic spiral bundle; RF, radial fibers; OC, organ of Corti. Bars indicate 100 μm.

In addition to the profound elongation of the radial bundles, the regular spacing and thickness of radial bundles was disrupted and there was a massive overshooting of many fibers beyond the organ of Corti (Fig. 5D, F). Labeling with anti-neurofilament and anti-tubulin primary antibodies followed by secondary antibodies tagged with different fluorophores, we next demonstrated nearly complete overlap of all labeled fibers in control animals (Fig. 6A, A’, A”). In contrast, several radial and nearly all fibers beyond the organ of Corti were in Sox10 mutants only labeled with anti-tubulin (Fig. 6B”, C”). Remarkably, and in contrast to neurofilament positive fibers, many tubulin positive fibers did not enter the organ of Corti but rather bypassed the organ to expand to the lateral wall (Fig. 6B, C). These fibers passed below the basilar membrane underneath the organ of Corti as previously reported for vestibular fibers rerouted into the cochlea after misexpression of neurotrophins [42] and in Foxg1 mutant mice [43]. With the exception of transient overshooting to the lateral wall in E16.5 ErbB2 null mice [7], such a profound and lasting projection to the lateral wall has never been reported before at this late stage in development.

Many tubulin-positive fibers followed the blood vessels and occasionally spiraled together with the spiral artery for some distance underneath the organ of Corti (Fig. 6B). Given the proximity of these fibers to blood vessels they could potentially be sympathetic autonomic fibers known to accompany blood vessels in the ear and elsewhere. However, autonomic ganglia are neural crest-derived and thus are absent in Sox10 mutants [22].

**Figure 7:**
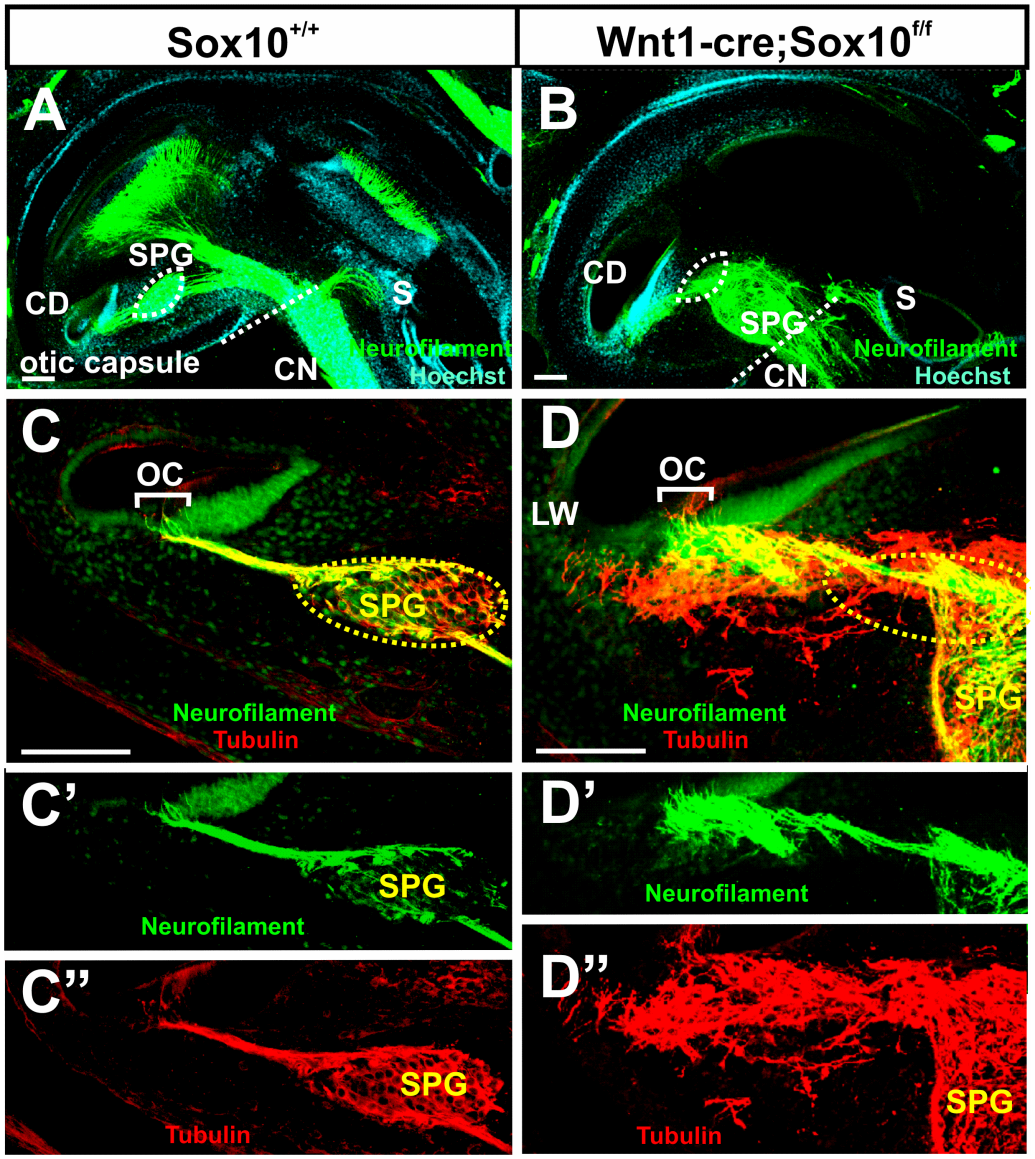
Immunochemistry of a coronally sectioned ear shows differences in spiral ganglion cell distribution and projection. Spiral ganglion neurons (SGN) are inside the otic capsule in the control ear (A) but have migrated to the center of the spiral and partially outside the ear in the Sox10 mutant (B, dotted line). The dotted circle in (A) indicates spiral ganglion location and is shown in (B) to indicate the position of Rosenthal’s canal. Most spiral ganglion neurons are tubulin positive and few are neurofilament positive in control (C, C’, C”) and Sox10 mutant mice (D, D’, D”). However, while all labeled fibers run together from spiral ganglion neurons to innervate hair cells in control mice (C, C’ C”), many tubulin labeled afferent fibers of Sox10 mutant mice bypass the organ of Corti (D, D’, D”). Abbreviations: OC, organ of Corti; HC, hair cells; LW, lateral wall. Bar indicates 100 μm.

We next wanted to rule out the possibility of differential penetration of antibodies in whole mounts as a reason for this unusual labeling profile of anti-tubulin immunostaining. We therefore investigated this unusual differential labeling in sections to sidestep such possible penetration problem. These sections confirmed the data generated in whole mounts and showed the unusual position of most spiral ganglion neurons in the center of the cochlea where control littermates have the cochlear nerve (Fig. 7A, B). The sections confirmed that some spiral ganglion neurons are in the Sox10 conditional mutants outside the otic capsule (Fig. 7B) whereas they are always near the cochlear duct in control littermates (Fig. 7A, B). Importantly, in the sections it was obvious that anti-tubulin labeled many spiral ganglion neurons whereas mostly efferent fibers of the intraganglionic spiral bundle were labeled with anti-neurofilament antibody (Fig. 7C, C’, C”, D, D’, D”). The sections demonstrate that it was mostly the anti-tubulin labeled fibers that are disorganized (Fig. 7D, D”) whereas anti-neurofilament stained fibers were mostly targeted to the organ of Corti, comparable to fibers in control littermates (Fig. 7C, C”).

Previous work showed that the labeling of efferent axons in the spiral ganglion, the intraganglionic spiral bundles (IGSB), is more profound using anti-neurofilament [44] consistent with our finding (Fig. 6A’, 7C’). These data suggest that many neurofilament-positive fibers entering the organ of Corti are efferent fibers. Additional fibers reaching outer hair cells in the basal region show patterns reminiscent of Type II afferents [44], [45].

Combined, our data imply that many spiral ganglion cells do not only migrate like vestibular ganglion cells but that their fibers have also lost the ability to navigate through the habenula perforata but rather remain on the scala tympani side of the organ of Corti, comparable to misrouted vestibular fibers [42]. Unfortunately, these data do not completely allow us to segregate efferents from afferents.

### Cochlear Afferents Extend to the Lateral Wall in Sox10 Conditional Mutant Mice

**Figure 8:**
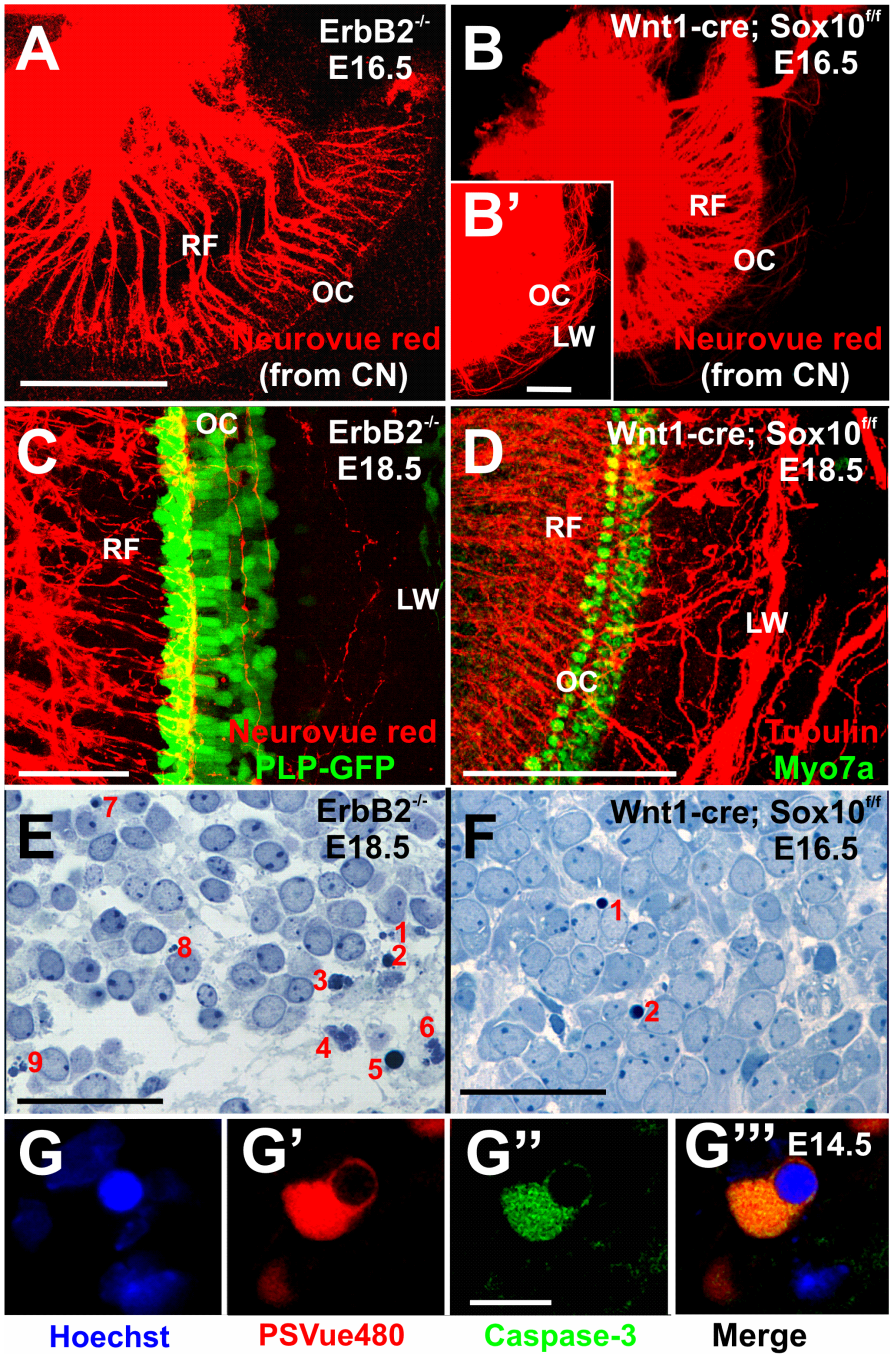
ErbB2 mutants with Sox10 conditional mutants have similar unusual spiral ganglion position but differ in fiber projections and apoptotic neuron ratio. Injection of lipophilic dyes into cochlear nuclei label spiral ganglion cells in the center of cochlea (A, B) and elongated radial fibers (RF). Only Sox10 mutants show a massive projection of spiral ganglion afferents to the lateral wall (insert in B). ErbB2 mutants have a severe reduction in radial fibers with very few extending into the organ of Corti (shown by PLP-eGFP) and the lateral wall (LW). In contrast, Sox10 mutants have a denser organ of Corti innervation (shown with Myo7a immunoreactivity in D) and many fibers to the lateral wall. One in 5 spiral ganglion neurons of ErbB2 mutants is apoptotic (E) and rare apoptotic profiles are also found in Sox10 mutants (F). Markers for apoptosis such as anti-activated caspase3 immunocytochemistry (G’) and PSVue (G”) confirm the occasional dying cell (G, G’”) in Sox10 conditional mutant mice. Abbreviations: LW, lateral wall; OC, organ of Corti; PLP-GFP, phospholipoprotein-green fluorescent protein; RF, radial fiber. Bar indicates 100 μm in A, B, D, 50 μm in C, E, F and 10 μm in G.

Using lipophilic dye tracing, we previously showed that selectively labeling of either afferents or efferents by insertion of these dyes into the efferent bundle and the cochlear nuclei, respectively [44], [45]. Using this approach we could label selectively afferents reaching to the cochlear nuclei (Fig. 8A). Increasing the gain of the images showed a dense meshwork of afferent fibers extending beyond the organ of Corti to the lateral wall (insert in Fig. 8B). These data indicate that without Schwann cells some afferents are so disoriented that they are unable to project into the organ of Corti.

In contrast, similar labeling in ErbB2 mutant mice showed fewer afferent fibers and hardly any afferents extending past the organ of Corti (Fig. 8A). Thus, while ErbB2 mutants and Sox10 conditional mutants show similar migratory effects of spiral ganglion neurons into the center of the cochlear spiral, there are differences in the density of afferent innervation of the organ of Corti and, most importantly, of overshooting afferent fibers present only in the Sox10 conditional mutant at this late stage. We next investigated this at the oldest age of viable mutants, E18.5 (Fig. 8C, D). In ErbB2 mutants we found only a few very thin fibers between outer hair cells and to the lateral wall (Fig. 8C). In contrast, Sox10 mutants showed a profound fiber growth to the lateral wall (Fig. 8D). Differences in density of innervation were also obvious with more radial fibers extending to the organ of Corti and the various hair cells in Sox10 mutants (Fig. 8D) compared to ErbB2 mutants (Fig. 8C). Therefore, while both mutants have disoriented fiber growth to the organ of Corti and beyond, this was accompanied by an overall reduction in fibers in ErbB2 mutant (Fig. 8C) whereas Sox10 conditional mutants showed massive extension of fibers to and past the organ of Corti (Fig. 8D).

**Figure 9:**
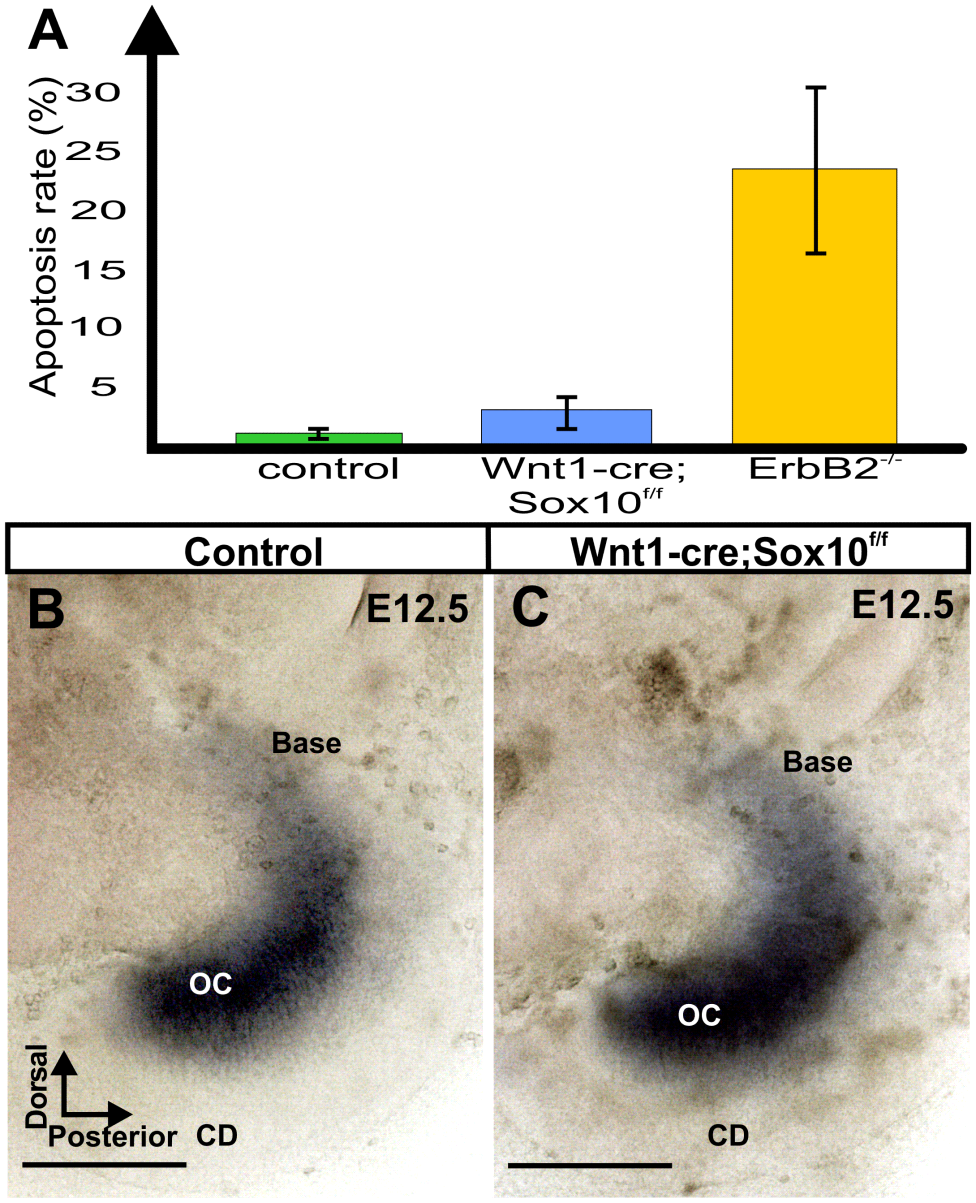
Conditional deletion of Sox10- with Wnt1-cre causes limited increase in apoptosis and no obvious neurotrophin expression change in the ear. Sox10 mutants and control littermates show limited apoptosis compared to ErbB2 mutants (A; E16.5–18.5 animals pooled). Previous work in ErbB2 mutants required q-PCR to show remaining NT-3 expression. This reduction of NT-3 correlated with the high level of apoptotic profiles in these mutants (A). In contrast, *in situ* hybridization for NT-3 shows similar distribution in control (B) and Sox10 conditional mutant (C) consistent with limited apoptosis (A). Abbreviations: CD, cochlear duct; OC, organ of Corti. Standard deviation is indicated by a vertical bar in (A). Bar indicates 100 um.

Given the reduction of fibers and previous observations of apoptotic profiles in ErbB2 mutants [7] we next compared the appearance of apoptotic profiles among inner ear sensory neurons (Fig. 8E, F) and verified that our identification of apoptotic cells is consistent with other markers of cell death such as activated caspase 3 and PSVue (Fig. 8G, G’, G”, G’”[34]). While both mutants showed apoptotic profiles, there was a noticeable difference in the apparent frequency of apoptotic profiles between the ErbB2 mutants (Fig. 8E) and Sox10 conditional mutants (Fig. 8F). We therefore counted normal sensory neurons and apoptotic profiles in separate sections at two stages of Sox10 conditional mutants, Sox10 control littermates and ErbB2 mutants (Table 1; Fig. 9A). We established a trend toward a difference in the frequency of apoptotic profiles in ErbB2 mutants compared to Sox10 conditional mutants. Even fewer apoptotic profiles were identified in control littermates, indicating a low frequency of programmed cell death as previously reported [31].

Previous work had linked the obviously elevated cell death in ErbB2 null mutants with a quantitative reduction in neurotrophin expression [7]. In fact, q-PCR was the only way to demonstrate NT-3 expression as in situ hybridization results did not show a signal in the mutant. Lower levels of neurotrophins might be related to the apparent increase in apoptosis in ErbB2 null mice, consistent with work demonstrating that NT-3 regulates survival of spiral ganglion neurons during development [46]. Indeed, the absence of NT3 and BDNF results in complete loss of all inner ear sensory neurons at birth [39]. Given the low frequency of apoptotic profiles in Sox10 conditional mutants (Fig. 8E, 9A), we hypothesized that neurotrophins should be near normally expressed in Sox10 conditional mutants and detectable by *in situ* hybridization. NT-3 expression was indistinguishable from control littermates prior to the onset of the cell death phase (Fig. 9B, C). Minor quantitative expression differences, not detectable by *in situ* hybridization, might be responsible for the somewhat elevated cell death of Sox10 conditional deletion mice compared to control littermates (Fig. 9A; Table 1).

### Loss of Sox10 Positive Cells does not Affect Central Projections

**Figure 10:**
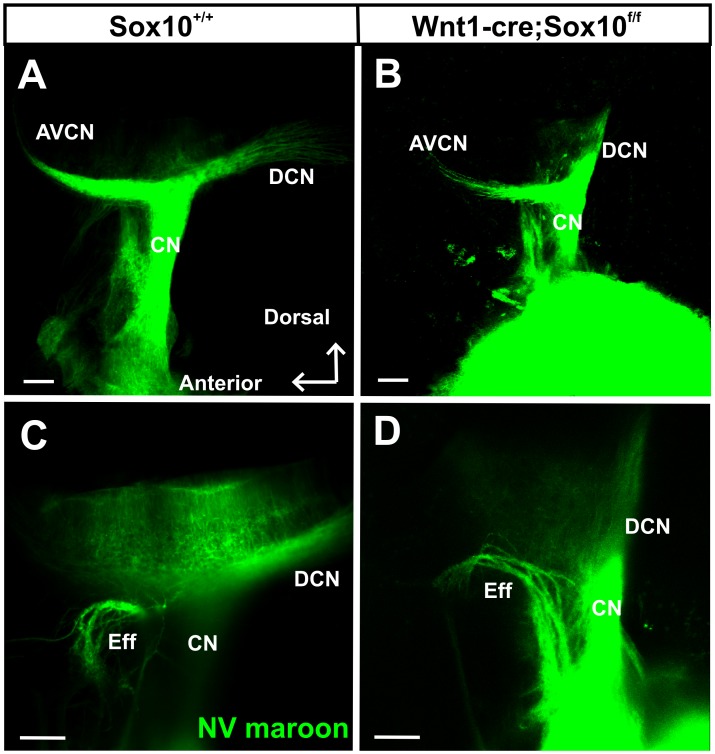
Conditional deletion of Sox10 does not affect the central projections of the cochlear nerve. Lipophilic-dye-soaked filter strips (NeuroVue maroon) were applied into the cochlear to label cochlear afferents to the cochlear nuclei (A) including the antero-ventral cochlear nucleus (AVCN) and dorsal cochlear nucleus (DCN). Cochlear afferents project normally in the Sox10 mutants (B). Olivocochlear efferent fibers (Eff) exit normally with the vestibular nerve root in the control and mutant mice(C, D). Abbreviations: CN, cochlear nerve. Bar indicates 100 μm.

Previous work showed minor disorganization of central vestibular projections in ErbB2 mutants [7], an observation that is difficult to interpret based on the widespread expression of ErbB2, not only in the Schwann cells, but also in glia cells in the central nervous system. We therefore wanted to investigate if there are any aberrant central projections or if spiral ganglion neurons can navigate to reach their target without neural crest-derived Sox10 positive cells. Our data (Fig. 10A, B) show that the central projections of control and Sox10 conditional mutants are indistinguishable at this or later stages (data not shown). Likewise, the efferent exit from the brain is normal (Fig. 10C, D). These data suggest that the minor effects in ErbB2 mutants are not related to the loss of Schwann cells.

### Loss of Sox10 Positive Cells Alters Vestibular Projections

**Figure 11:**
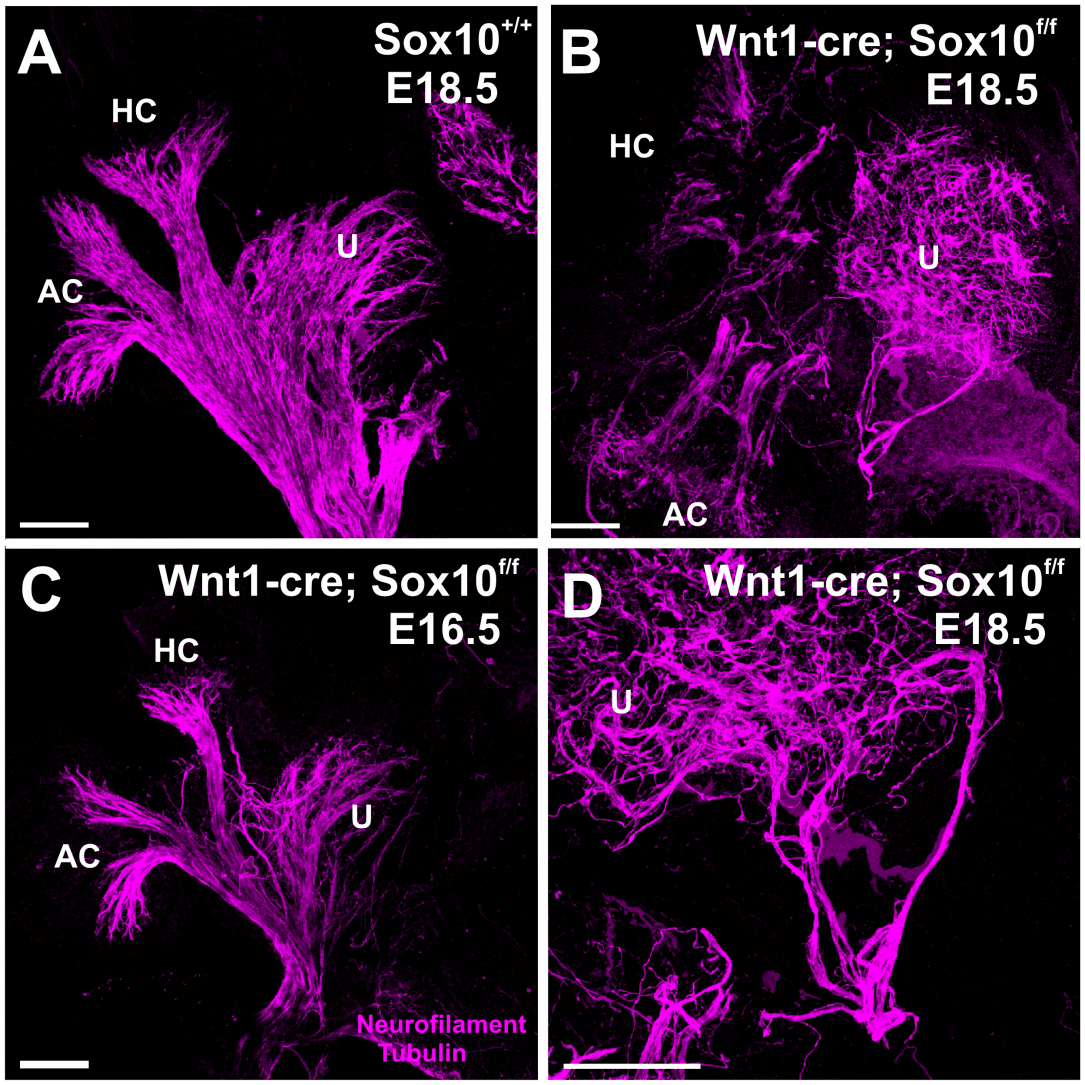
Afferents to vestibular epithelia are reduced and disorganized. Neural crest-derived Sox10 positive cells play a guidance function in the vestibular innervation of the ear. Compared with control mice of E18.5 (A), Sox10 conditional mutants show a reduced innervation to the canal cristae(B, D) and disorganized fibers routing from one canal crista to another and occasionally to nowhere (B, D). Note the smaller size of epithelia and fiber projections in the younger Sox10 mutant (B). Abbreviations: PC, posterior crista; AC, anterior crista; HC, horizontal crista; U, utricle. Bar indicates 100 μm.

Mice with a conditional deletion of Sox10 retained a reduced innervation to the canal cristae (Fig. 11A, B) and utricle (Fig. 11, C, D). The defects were overall comparable to those reported for ErbB2 null mutants [7]. In contrast to the organ of Corti there was no massive overshooting of fibers beyond the hair cells of the vestibular sensory organs, indicating that overall targeting of sensory epithelia is less affected in vestibular sensory epithelia (Fig. 11) compared to the organ of Corti (Fig. 6–8). These data suggest that presence of neural crest-derived Sox10 positive cells (or ErbB2 positive cells) plays at best a limited role in guiding the vestibular innervation of the ear.

**Figure 12:**
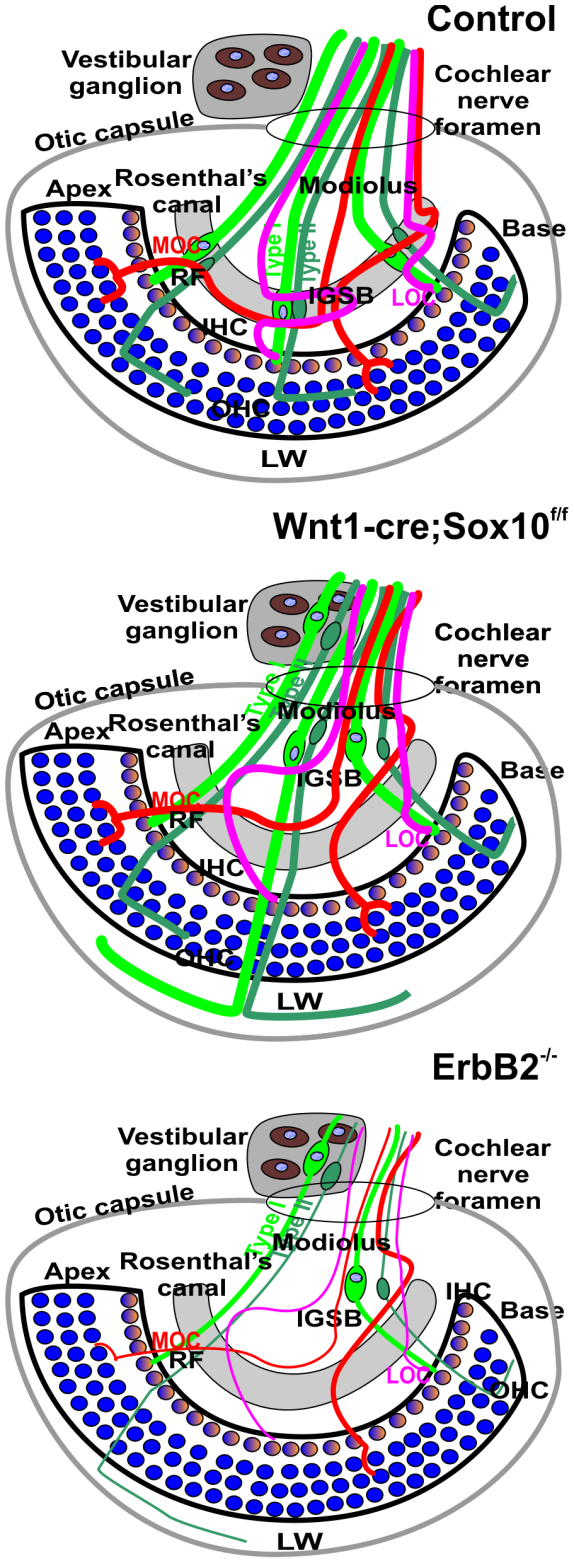
Schematic depiction of the major effects of loss of Schwann cells in Sox10 conditional deletion mutants and ErbB2 mutants. The single row of inner hair cells (IHC) is innervated by type I afferents, the three rows of outer hair cells (OHC) are innervated by type II afferents. All spiral ganglion neurons are in parallel to the organ of Corti in Rosenthal’s canal (grey). In addition, two types of efferent fibers (Medial and Lateral Olivocochlear fibers; MOC, LOC) form intraganglionic spiral bundles (IGSB) and join the afferents in radial fibers (RF) to the organ of Corti. Conditional deletion of Sox10 or ErbB2 leads to excessive migration of spiral ganglion neurons beyond Rosenthal’s canal into the modiolus that normally carries only afferent and efferent fibers. Some spiral ganglion neurons leave the ear to intermingle with vestibular neurons that always migrate outside the otic capsule. Instead of forming an IGSB, efferent fibers crisscross randomly along the greatly lengthened radial fibers. Many, but not all afferents, bypass the organ of Corti and extend to the lateral wall (LW). The overall pattern of migration and disorganization of fibers is similar in ErbB2 mutants. However, at E18.5 there are hardly any fibers to the lateral wall left in ErbB2 mutants; fewer fibers reach the organ of Corti and those that do are very thin. It appears that elimination of Schwann cells by either Sox10 conditional deletion or ErbB2 mutation has an overall similar phenotype of excessive migration and overshooting afferent projection. Differences seem to relate to additional effects of ErbB2 on NT3 expression and are not specific to Schwann cells. Blue indicates BDNF expression in hair cells, yellow indicates NT3 expression in inner hair cells. Abbreviations: IGSB, intraganglionic spiral bundles; IHC, inner hair cells; OHC, outer hair cells; RF, radial fibers; MOC, medial olivocochlear fibers; LOC, lateral olivocochlear fibers; LW, lateral wall.

In summary, our data on conditional deletion mutants of Sox10 using Wnt1-cre show profound defects in the inner ear innervation that range from migration defects of neurons to novel projections of spiral ganglion cells to the lateral wall (Fig. 12). Our data demonstrate that the interaction of inner ear-derived sensory neurons with neural crest-derived Schwann cells provides guidance to the peripheral, but not the central projection of inner ear sensory neurons.

## Discussion

Sox10 is crucial for neural crest-derived cells to develop into Schwann cells, melanocytes, enteric neurons and various autonomic and sensory ganglia [14], [16], [47]. We show here that the conditional deletion of the floxed Sox10 gene [12] with neural crest-specific Wnt-1-cre [26], [28] leads to loss of Schwann cells in the entire developing inner ear ganglion. Conditional loss of Sox10 with Wnt1-cre does not affect Sox10 expression in the sensory epithelia of the ear or the normal differentiation of inner ear sensory neurons and epithelia. A dual origin of inner ear neurons and hair cells from both the inner ear and neural crest was recently suggested [36]. This claim is difficult to reconcile with data generated by knocking out an ear specific expression of *Gata3* using the ear specific expression of *Foxg1* that eliminates all neurosensory cells of the cochlea [48]. Likewise, most recent tracing data [20] using Wnt1-cre are more consistent with the traditional claims [3], [19] that all neurosensory cells are derived from the ear. Our own data using Wnt1-cre to eliminate floxed Sox10 are more consistent with the most recent data on this subject [20] but additional work is needed to refute the earlier claim of a dual origin of neurosensory cells [36]. We show here is that the absence of neural crest-derived Schwann cells leads in turn to abnormal migration of spiral ganglion neurons into the center of the cochlea and aberrant projections of afferents past the organ of Corti into the lateral wall of the cochlea (Fig. 8D, 12).

### Migration Defects

Spiral ganglion neurons in the mouse ear become postmitotic between E10.5 and E12.5, and migrate from the cochlear duct to Rosenthal’s canal [49], [50]. At or even shortly before delamination from the ear these neurons project axons toward the brain [39], [51] and translocate within these leading processes. Concomitant with settling in Rosenthal’s canal these neurons project radial fibers to the organ of Corti (Fig. 12). While this process is overall identical in vestibular and spiral ganglion neurons, there is a distinct differential distribution of these two cell types. Vestibular ganglion neurons migrate to become Scarpa’s ganglion between the ear and the brain and project to the five vestibular sensory organs peripherally and the vestibular nuclei centrally [52], [53]. In contrast, spiral ganglion neurons remain entirely inside the ear and form a ganglion that co-extends with the spiraling cochlear duct, remaining at nearly uniform distance to the organ of Corti. In ErbB2 null mice [7] and in Sox10 conditional deletion mice there are no differentiated Schwann cells and the migration of spiral ganglion neurons extends into the modiolus and even outside the ear (Figs. 3, 7, 12), mixing with vestibular neurons (Fig. 3). These data suggest that Schwann cells or other neural crest-derived cells provide a stop signal for the migratory spiral ganglion neurons [39]. It is possible that the unique gene expression profile associated with spiral ganglion neurons compared to vestibular ganglion neurons [48] provide for a distinctly different interaction with uniform Schwann cells in the evolutionary new spiral ganglion neurons of mammals [54]. However, it is also possible that the Schwann cells in the cochlear nerve are different as this is the only nerve root where central neurons are known to migrate into the proximal cochlear nerve to form the so-called ‘root neurons’ [55], blurring the distinction between CNS and PNS.

It is possible that Schwann cell signal is biochemically related to the central nervous system stop signal of oligodendrocytes (Nogo) that blocks neurite extension in spinal cord regeneration [56]. Recently the interaction of Robo/Slit was shown to play a role in spiral ganglion migratory process [57]. However, given the more profound migratory defects shown in our Sox10 mutants and in ErbB2 null mutants [7] spiral ganglion neurons it is obvious that this interaction with Robo/Slit plays only a minor role and cannot be the entire stop signal for spiral ganglion neurons to end in Rosenthal’s canal (Fig. 12). A detailed comparison of the recently published expression profile of spiral ganglion cells [58] with other guidance cues identified in the developing ear [59] could help narrow down the candidates for the stop signal that is provided by the interaction between neural crest-derived cells and spiral ganglion cells. This could offer a molecular understanding how spiral ganglion neurons settle in Rosenthal’s canal in normal development.

### Loss of Sox10 Dependent Cells Causes Only Minor Additional Cell Death

Previous work on ErbB2 mutant mice showed a similar phenotype of excessive migration of spiral ganglion cells and transient overshooting projection to the lateral wall of the cochlea [7]. However, these fibers, present only in some E16.5 day old ErbB2 null mice, had nearly all disappeared by E18.5 (Fig. 8A, C). We suggest that the loss of these fibers in ErbB2 mutants might be correlated with the excessive neuronal death (Fig. 8E), presumably related to the downregulation of neurotrophins in the ear demonstrated with q-PCR. Our data show an even more profound outgrowth of fibers to the lateral wall (Fig. 5–7, 8A, C) of Sox10 conditional mutant mice. In contrast to ErbB2 mutants, many of these fibers persist at least until E18.5 (Fig. 6,7) in Sox10 conditional mutants, suggesting that little correction of their projection errors happen. Correction of projection errors was an idea that led to the identification of neurotrophins as the molecular means to do such corrections [60]. The major neurotrophin factor in the developing cochlea is NT-3 [46], [61], [62]. We therefore verified that the expression of this neurotrophin was not grossly different from control littermates (Fig. 9), suggesting that sparing Sox10 expression in the ear leaves neurotrophin expression nearly normal.

Using various techniques [34] we confirmed that the histologically detectable apoptotic cells have characteristics of dying cells (Fig. 8G). Consistent with the limited changes in NT-3 expression is the rather sporadic appearance of apoptotic cells in Sox10 conditional deletion mice compared to ErbB2 mutant mice (Fig. 8E, F). Quantifying the apoptotic cells showed a trend towards a difference between these mutants (Fig. 8F, G; 9A). These data indicate that not a profound change of NT-3 expression but other mechanisms may account for the limited spiral ganglion neuron degeneration in the conditional Sox10 mutant mice. Our data on conditional Sox10 mutants (Fig. 8E, F; 9) demonstrate for the first time that absence of Schwann cells has on its own a very limited effect on embryonic spiral ganglion neuron viability. This is markedly different to the absence of neurotrophins [39], [61], neurotrophin receptors [63], hair cells [41], [64], or neurotrophin downregulation in ErbB2 mutants [65].

### Schwann Cells Provide Guidance for Spiral Ganglion Cell Afferents to Enter the Organ of Corti

Previous work by others has provided evidence in favor of and against a role for neural crest-derived cells in nerve fiber guidance. This work can be categorized into two groups: experimental manipulations (microsurgery in chicken) and molecular manipulations (mostly in mice, zebrafish and frogs). Experimental manipulations tend to support that the continuity between the developing placodes and the brain provide for proper fiber growth [4], [5]. Many of these manipulations of neural crest ablation always disrupt mesenchymal cell bridges that could provide guidance cues for both neural crest-derived cells and placode-derived neurons. Using Wnt1-cre induced diphtheria toxin-mediated neural crest ablation would leave such mesenchymal cell bridges intact and indicates no guidance defect [8]. Clearly, absence of Schwann cells does not affect the central projection (Fig. 10), known to be affected by mutations of genes expressed in the neurons [66], but derails afferent projections to the cochlea (Fig. 8A, C).

Our data concur with other Schwann cell ablation studies that indicate that the directed growth of peripheral processes of spinal nerves and the inner ear depend on normal development of Schwann cells [6], [7]. In fact, the defect reported here in the otherwise stereotyped organization of radial fiber bundles exceeds any other mutational defect reported thus far, except for the profound disorganization reported in delayed conditional deletion of Gata3 [67]. These defects on delayed Gata3 conditional deletion are more profound than either the very limited fiber disorganization reported in mesenchyme mutants [68] or the disorganization after loss of Schwann cells we report here. It remains unclear how the loss of Gata3 mediated guidance defects can be reconciled with either of these two previously identified guidance principles (mesenchyme, Schwann cells).

Despite the profound disorganization reported in conditional Gata3 mutants, targeting of the organ of Corti was near normal [67]. In contrast, Sox10 conditional mutants and some other mutants [43] have overshooting fibers into the lateral wall also reported transiently in ErbB2 mutants [7]. Somewhat similar disorganization of afferents exists in mutants misexpressing neurotrophins. In fact, the extension along the spiral arteries in neurotrophin misexpressing mutants [39] is very reminiscent of the major fiber tracts we find here in Sox10 conditional mutants. Given that all the aberrant fibers in neurotrophin misexpressors [39] are rerouted vestibular neuron processes that follow the misexpressed BDNF into the cochlea, it is noteworthy that spiral ganglion neurons of the Sox10 conditional mutants mix with vestibular neurons (Fig. 3) and project much like rerouted vestibular neurons [42]. Absence of Schwann cells changes both spiral ganglion neuron migration and peripheral, but not central cochlear nucleus projections, to adopt a vestibular neuron-like phenotype. Both rerouted vestibular fibers [42] and spiral ganglion neurons in conditional Sox10 mutant mice may follow other cues such as blood vessels, normally avoided due to the preferred guidance cues provided by Schwann cells.

### Conditional Absence of Sox10 Shows Limited Cell Death

Loss of ErbB2 results not only in loss of Schwann cells but also in reduced levels of neurotrophin expression and massive cell death [7]. Consistent with this effect, earlier work on inner ear development claimed reduction of the cochlea in Sox10 mutants [9], possibly through the regulation of Jagged 1. Since a shortened cochlear duct could reduce the availability of neurotrophins released from the hair cells and supporting cells, one would expect an increase of neuronal death in these mutants. However, in a follow up paper it was reported that there is no effect on neuronal death despite the shortening of the cochlea and the absence of Schwann cells [10]. In contrast to this claim, our own data show a very early and profound effect on cochlear innervation but no obvious effect on cochlea development (Fig. 5A, B). Moreover, simple elimination of Schwann cells in our neural crest specific deletion of Sox10 results in no obvious alteration of neurotrophin expression and limited neuronal death (Fig. 8F; Table 1). This suggests that other neurotrophic factors in part generated by Schwann cells and invoked in postnatal survival of spiral ganglion neurons [69] have little effect on embryonic spiral ganglion neurons. Unfortunately, our model does not allow studying postnatal stages and the mixing of spiral and vestibular ganglion neurons precludes a deeper quantitative assessment.
